# Racial Disparities in U.S. Peripartum Cardiomyopathy: Systematic Review and Meta-Analysis of Risk Factors and Outcomes

**DOI:** 10.1016/j.jacadv.2026.102653

**Published:** 2026-03-16

**Authors:** Sarah P. Hermans, Alexander N. Arreguin, Jianing Ma, Maged M. Costantine, Xueliang Pan, Lauren J. Hassen

**Affiliations:** a Department of Internal Medicine, The Ohio State University Wexner Medical Center, Columbus, Ohio, USA; b Center for Biostatistics, College of Medicine, The Ohio State University Wexner Medical Center, Columbus, Ohio, USA; c Department of Obstetrics & Gynecology, Division of Maternal-Fetal Medicine, The Ohio State University Wexner Medical Center, Columbus, Ohio, USA; d Department of Internal Medicine, Division of Cardiovascular Medicine, The Ohio State University Wexner Medical Center, Columbus, Ohio, USA

**Keywords:** peripartum cardiomyopathy, postpartum cardiomyopathy, pregnancy-associated cardiomyopathy, racial disparities

## Abstract

**Background:**

Peripartum cardiomyopathy (PPCM) is a leading cause of heart failure in pregnancy and contributes significantly to maternal morbidity and mortality. Black women are disproportionately affected and experience worse outcomes compared with other groups.

**Objectives:**

This study aimed to quantify differences in risk factors and outcomes between Black and White women in the United States diagnosed with PPCM.

**Methods:**

We conducted a systematic review and meta-analysis of observational studies published after 2002 including U.S. women with PPCM and race-stratified risk factors and outcomes. Investigated outcomes included mortality, major adverse cardiac events, and recovery of left ventricular ejection fraction. Random-effects meta-analysis estimated the pooled prevalence of risk factors and outcomes. Logistic regression, forest plots, and I^2^ statistics were utilized for analysis.

**Results:**

Compared with controls, cases had higher rates of obesity, preeclampsia, hypertension, diabetes, multiple gestations, and tobacco use. Compared to White cases, Black cases had higher prevalence of diabetes (14% vs 5%; *P* = 0.027) and utilization of public payer (72% vs 30%; *P* < 0.001). At presentation, mean left ventricular ejection fraction was 26% in Black women and 29% in White women. White women experienced higher rates of recovery in ejection fraction (63% vs 40%; *P* < 0.0001). Mortality rates were higher among Black women (8% vs 2%; *P* = 0.013).

**Conclusions:**

Black women with PPCM experienced lower recovery and higher mortality rates compared with White women. With the exception of a significant difference in payer status, modest differences in previously identified risk factors were observed between racial groups to account for worse outcomes (Disparities in risk factors and outcomes between Black and White US women with peripartum cardiomyopathy: A systematic review and meta-analysis; CRD42023439228).

Peripartum cardiomyopathy (PPCM) is a common cause of heart failure during pregnancy and postpartum, and a significant contributor to maternal morbidity and mortality. It is defined by the onset of heart failure with a left ventricular ejection fraction (LVEF) of <45%, occurring late in pregnancy or within 5 months postpartum in women without prior cardiovascular disease.^1^ Incidence varies across geographic regions and ethnicities (1 in 100-1 in 15,500 live births),^2^ and 1-year mortality ranges from 4% to 14%.^2^^,^^3^ Notably, Black women are disproportionately affected and experience worse outcomes compared to other racial groups.^4^ In U.S. cohorts, the incidence of PPCM is 2.7 to 4.6 times higher in Black women compared to White women.^4^, ^5^, ^6^, ^7^, ^8^, ^9^, ^10^ Black women with PPCM more often present with severe symptoms of heart failure, exhibit higher mortality rates, and have slower recovery of EF compared to White patients.^11^, ^12^, ^13^ In Black women with recovered EF, the time to recovery was twice as long as in other groups.^12^

These disparities have been attributed to genetic variants, autoimmune disorders, angiogenic imbalance, pre-existing conditions, variations in disease pathophysiology, socioeconomic disparities, and access to care.^14^ Additional factors may include a higher incidence of risk factors for PPCM, such as hypertensive disorders of pregnancy, multiple gestations, and diabetes.^9^^,^^14^ Differences in the prevalence of these risk factors between Black and White women diagnosed with PPCM have been inconsistent across research. Several studies have suggested that the increased risk in Black American women is independent from sociodemographic factors and differences in medical treatment.^4^^,^^11^^,^^15^

Observational studies have identified disparities in PPCM incidence, morbidity, and mortality between racial groups within the United States. However, to our knowledge, no systematic review and meta-analysis has been conducted to analyze disparities in risk factors and outcomes related to PPCM in Black American women compared with other racial groups in the United States. This study aims to quantify differences in known risk factors between Black and White populations and to gain further understanding behind disparities in outcomes.

## Methods

### Literature and data search

This review followed the Preferred Reporting Items for Systematic Reviews and Meta-Analyses guidelines and was registered with International Prospective Register of Systematic Reviews (CRD42023439228).^16^ The full protocol, as previously published, is included in Supplemental Appendix A. We utilized databases including EMBASE, MEDLINE, Web of Science, and Google Scholar; screened reference lists and meta-analyses, and conducted gray-literature searches (Networked Digital Library of Theses and Dissertations; Google Scholar).^17^ Searches included English language articles published after January 1, 2002. Institutional Review Board and ethical approval were not required for secondary analysis of published, deidentified aggregate data.

### Literature screening, study selection, and eligibility criteria

Two reviewers (S.H., A.A.) screened titles and abstracts using Covidence, compared references, and obtained full texts.^18^ Full texts were screened, and reasons for exclusion were recorded. For studies with data overlap, the study with the largest sample size was included.

**Figure 1:**
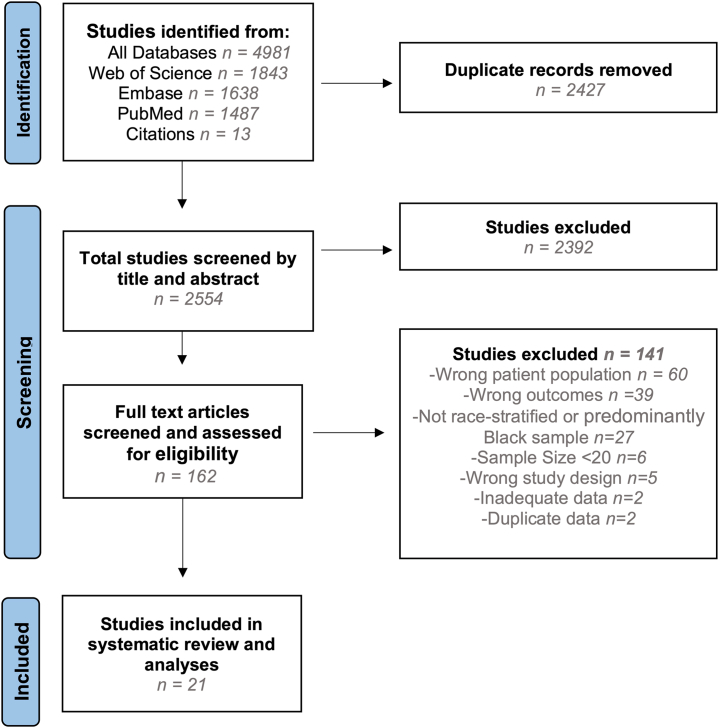
**PRISMA Flow Diagram of Study Selection** Diagram demonstrating identification, screening, eligibility assessment, inclusion, and exclusion of studies examining peripartum cardiomyopathy outcomes by race.

Inclusions were limited to primary observational studies. Figure 1 illustrates reasons for exclusion.

Participants included women with a diagnosis of PPCM consistent with NHLBI/ORD is the National Heart, Lung, and Blood Institute and the National Institutes of Health Office of Rare Diseases Workshop definition or categorized by ICD-9-CM 674.50 to 55, or ICD-10-CM O90.3.^1^ Participants who were diagnosed with PPCM in prior pregnancies were excluded.

Included studies reported data from predefined risk factors for PPCM stratified by race or belonging to a majority Black population AND/OR studies that included outcomes stratified by race or belonging to a majority Black population. Most studies used electronic medical record data in which race was self-reported. Other ethnic groups were not included due to lack of prior research and introduction of heterogeneity.

### Data extraction

Extracted data included study characteristics (investigator, title, journal, years, sample source, study type, location, definition of PPCM, race stratification, and follow-up period) and risk factors of PPCM (age >30 years old, obesity, preeclampsia, gestational hypertension, chronic hypertension, diabetes, gestational diabetes, multiple gestations, tobacco use, and payer). The primary outcome investigated included mortality; secondary outcomes included EF recovery and major adverse cardiac outcome.

### Assessment of methodological quality

Two reviewers assessed bias through use of the Newcastle–Ottawa Scale in Supplement Appendix B.^19^

### Meta-analytic approach and statistical analysis

A descriptive analysis was conducted for each study. The number of patients with risk factors and outcomes was extracted and stratified by race and case status. Statistical analysis was conducted using R software (α = 0.05).^20^ Research has demonstrated variation in incidence of PPCM-related risk factors by race. In cases, random-effects meta-analysis of proportions was conducted to estimate the pooled prevalence of each risk factor and outcome as race-specific data was present across multiple studies for each variable. Conversely, race-specific data on risk factors prevalence in controls were frequently limited to a single study; therefore, pooled prevalence was estimated via a fixed-effects model without race adjustment. A statistical comparison between the 2 pooled estimates was not conducted, as it would assume comparable underlying racial distributions in both groups. Logistic regression was used to investigate the association between each variable (risk factors and outcome) and races (Black vs White), groups (PPCM vs control), and their interaction. Covariate adjustment in the regression model was not performed due to the use of aggregate data rather than individual data, as well as heterogeneity and variable reporting. Statistical heterogeneity was estimated via I^2^ statistics. Medication utilization between cases was additionally investigated; differences between racial groups (*P* values) were reported when at least 1 study was available in each race. Race-specific pooled estimates were only calculated when 2 or more studies were available for that subgroup. Results shown for only Black participants in table 5 indicate that only 1 study contributed data for White participants for that risk factor.

## Results

### Study selection

Figure 1 demonstrates a PRISMA diagram of study selection and inclusion. Ultimately, 23 met the inclusion criteria, but 2 were later excluded for duplicate data. Supplemental Tables in Appendix D describes included studies and populations. In total, 12,502 cases were included: 6,090 (48.7%) self-identified as Black and 6,412 (51%) as White. For Black patients, data were extracted from 14 race-stratified studies (5,820, 96%) and 7 studies (270 cases, 4%) in which race was not stratified but were majority Black cohorts (ie, >74%). For subjects self-identified as White, data were extracted from 8 race-stratified studies including 6,067 (95%) of subjects; 345 (5%) were not stratified but were predominately White cohorts with minimal prevalence of Asian/unknown/Hispanic cases. Four studies included 13,068,886 controls. Two studies were race-stratified, totaling 1,768,465 participants, 324,948 classified as Black and 1,443,517 as White. Of the 21 studies, 5 studies including 11,163 (89%) cases had no follow-up and included data limited to admissions; most limited to delivery admissions and 1 included admissions postdelivery. Of studies with follow-up, follow-up spanned 6 to 76 months with most recovery assessments completed within 6 to 12 months.

### Prevalence of PPCM-related risk factors in cases vs controls and racial differences in risk factor distribution

#### Cases vs controls

**Figure 2:**
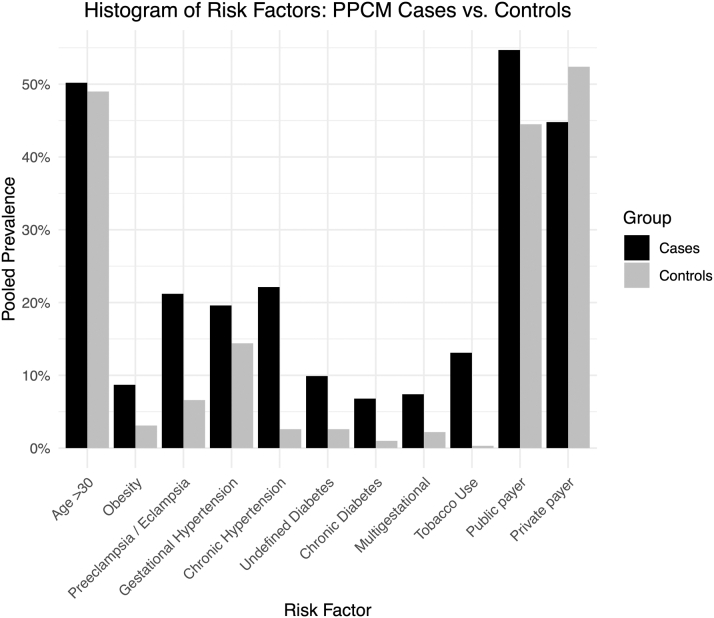
**Histogram of Risk Factors: Peripartum Cardiomyopathy Cases vs Controls** Comparison of cardiovascular and obstetric risk factor prevalence between peripartum cardiomyopathy cases and healthy controls. PPCM = peripartum cardiomyopathy.

Compared to controls, cases had higher prevalence of most risk factors (Table 1, Figure 2). The largest differences included prevalence of preeclampsia (21% [95% CI: 15%-29%] vs 7% [95% CI: 4%-12%]), chronic hypertension (22% [95% CI: 14%-34%] vs 3% [95% CI: 0.4%-15%]), and undefined diabetes (10% [95% CI: 6%-16%] vs 3% [95% CI: 1.0%-6.77%]). Undefined diabetes mellitus included chronic or pregestational diabetes and gestational diabetes, as well as cases in which the studies did not specify timing of diagnosis. Prevalence of age >30 years, gestational hypertension, and payer status was comparable between groups.

**Table 1:** Random-Effects Model Comparison of Risk Factors in Cases, Controls, and Race-Stratified Cases

	Overall Cases				Black PPCM			White PPCM			*P* Value	Overall Control			
	N	Studies	%	95% CI	n	%	95% CI	n	%	95% CI		N	Studies	%	95% CI
Age >30 y	686	4	50.2	39.5-60.9	310	39.5	33.2-46.1	376	63.8	56.7-70.4	<0.001	1,766,087	1	49	49.0-49.1
Obesity	10,335	3	8.7	5.8-12.7	5,006	11.6	10.8-12.6	5,329	7.9	4.3-14.3	0.208	13,066,424	3	3.1	1.3-6.8
Preeclampsia/Eclampsia	11,309	7	21.2	15.0-29.3	5,429	19.2	12.3-28.6	5,880	24.2	13.8-38.9	0.498	13,068,802	3	6.6	3.6-11.7
Gestational hypertension	11,163	6	19.6	8.3-39.5	5,352	19.6	5.8-49.1	5,811	19.5	5.6-49.9	0.995	11,302,715	2	14.4	7.2-26.7
Chronic hypertension	11,748	9	22.1	13.8-33.5	5,595	28.0	16.8-42.6	6,153	15.7	6.6-32.9	0.217	13,062,413	2	2.6	0.4-14.5
Undefined diabetes	10,729	10	9.9	6.1-15.7	5,285	13.9	8.6-21.6	5,444	5.0	2.2-10.8	0.027	13,066,508	4	2.6	1.0-6.7
Chronic/pregestational diabetes	10,621	7	6.8	3.7-12.3	5,177	10.0	5.3-18.2	5,444	3.9	1.4-10.3	0.113	13,066,424	3	0.9	0 0.7-1.1
Multigestational	4,855	7	7.4	3.9-13.5	2,372	6.4	2.4-15.9	2,483	8.9	4.4-17.4	0.576	1,170,098	2	2.2	2.0-2.4
Tobacco use	4,692	8	13.1	6.4-24.8	2,318	12.6	4.1-33.1	2,374	13.0	5.7-27.1	0.964	1,772,560	3	0.3	0.02-5.3
Public payer	417	3	54.7	30.4-77.0	195	72.5	55.1-85.0	222	30.2	24.5-36.5	<0.001	13,066,424	3	44.5	31.4-58.4
Private payer	417	3	44.8	22.8-69.1	195	27.3	15.1-44.2	222	70.3	58.5-79.9	<0.001	13,066,424	3	52.4	38.8-65.7

PPCM = peripartum cardiomyopathy.

#### Cases stratified by race

**Figure 3:**
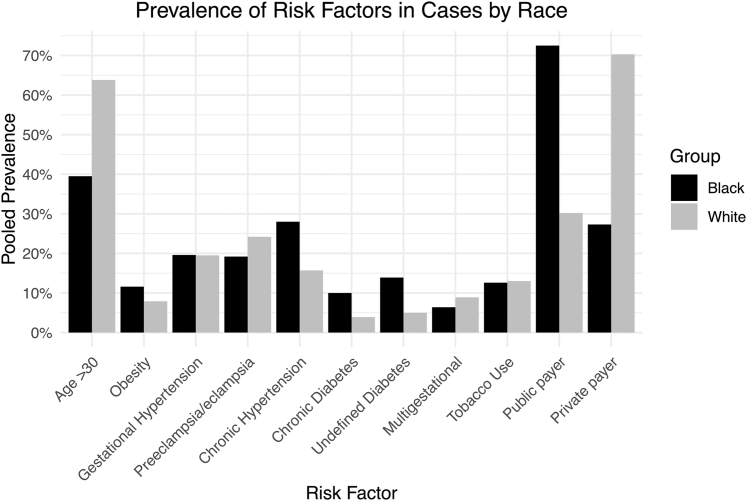
**Prevalence of Risk Factors in Peripartum Cardiomyopathy Cases by Race** Comparison of cardiovascular and obstetric risk factor prevalence among Black and White women with peripartum cardiomyopathy.

Table 1 and Figure 3 illustrate differences in risk factors between cases by race. White cases had a higher prevalence of presentation at age >30 years (64% [95% CI: 57%-70%] vs 40% [95% CI: 33%-46%]; *P* < 0.001). There was no statistically significant difference in the prevalence of preeclampsia (24% [95% CI: 14%-39%] vs 19% [95% CI: 12%-29%]; *P* = 0.498), chronic hypertension (16% [95% CI: 7%-33%] vs 28% [95% CI: 17%-43%]; *P* = 0.217), or obesity (8% [95% CI: 4%-14%] vs 12% [95% CI: 11%-13%]; *P* = 0.208). Undefined diabetes mellitus had a higher prevalence in Black cases (14% [95% CI: 9%-22%] vs 5% [95% CI: 2%-11%]; *P* = 0.027). There was no significant difference in pregestational diabetes or multifetal gestation.

There were significant differences observed in insurance coverage among cases. Black women were more likely to utilize a public payer (73% [95% CI: 55%-85%] vs 30% [95% CI: 25%-37%]; *P* < 0.0001). Logistic regression was conducted to investigate the effect of risk factors on PPCM odds by race. Results signified differences in risk factor prevalence by race between case and control populations. Obesity (interaction OR: 0.79; 95% CI: 0.69-0.90; *P* < 0.001), preeclampsia/eclampsia (interaction OR: 0.41; 95% CI: 0.36-0.46; *P* < 0.001), gestational hypertension (interaction OR: 0.728; 95% CI: 0.561-0.947; *P* = 0.017), tobacco use (interaction OR: 0.752; 95% CI: 0.631-0.896; *P* = 0.001), and multiple gestations (interaction OR: 0.544; 95% CI: 0.397-0.736; *P* < 0.001) demonstrated a stronger association with PPCM in White women compared to Black women. Diabetes (undefined diabetes interaction OR: 1.300; 95% CI: 1.087-1.559; *P* = 0.004; chronic diabetes interaction OR: 1.663; 95% CI: 1.340-2.076; *P* < 0.001) showed increased association with PPCM for Black women compared to White women. Age >30 years and chronic hypertension showed similar effect on PPCM risk between racial groups.

### Racial differences in rates of PPCM-related recovery, adverse cardiac events, and mortality

**Figure 4:**
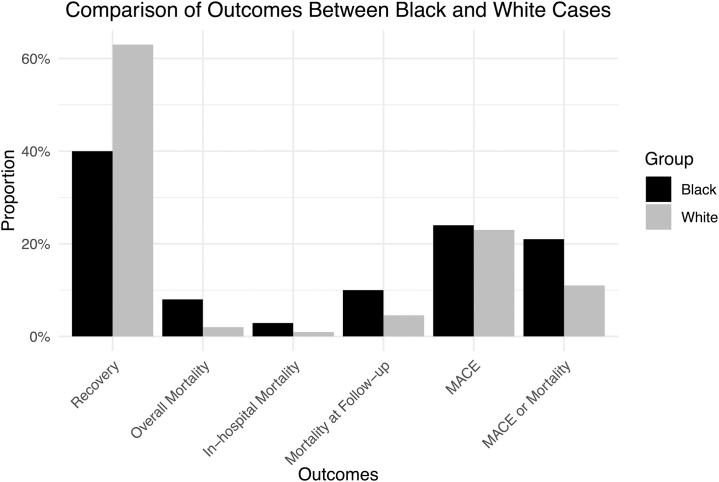
**Comparison of Outcomes Between Black and White Peripartum Cardiomyopathy Cases** Comparison of clinical outcomes in Black vs White women with peripartum cardiomyopathy.

The primary outcome investigated was mortality from any cause. Secondary outcomes included EF recovery and major adverse cardiac events (MACE) (Tables 2 and 3, Figure 4). Differences in rates of Caesarean deliveries and postpartum presentations were also investigated (Table 4). Caesarean delivery rates were similar in Black and White cases (53% [95% CI: 44%-63%] vs 61% [95% CI: 53%-69%]; *P* = 0.216). The proportion of patients presenting postpartum was also similar (84% [95% CI: 76%-90%] vs 84% [95% CI: 70%-92%]; *P* = 0.998). Baseline EF was lower in Black (mean EF, 26% [range: 18%-31%]) compared with White cases (mean EF, 30% [range: 28%-36%], *P* = 0.049). At follow-up, there was a trend toward greater improvement in EF in White (mean EF, 46% [range 36% to 56%]) compared with Black cases (mean EF, 38% [range: 32%-47%]), but this was not statistically significant (*P* = 0.062).

**Table 2:** Baseline and Follow-Up Ejection Fraction Comparison in Cases

	Black PPCM			White PPCM			*P* Value
	n	Mean	Min-max	n	Mean	Min-max	
Baseline EF	517	26.2	18-31	392	29.9	27.6-36	0.049
Follow-up EF	344	38.2	32-47	343	45.5	36.3-56.0	0.062

EF = ejection fraction; other abbreviation as in Table 1.

**Table 3:** Random-Effects Model Comparison of Outcomes in Cases

	Black PPCM			White PPCM			*P* Value
	n	%	95% CI	n	%	95% CI	
Recovery	605	40.1	34.1-46.4	455	62.8	54.0-71.0	<0.001
Overall mortality	2,288	8.4	5.3-12.9	1,952	2.4	0.97-5.8	0.013
In-hospital mortality	1,826	2.9	2.2-3.8	1,774	1.4	0.1-12.1	0.540
Mortality at follow-up	567	10.3	6.8-14.5	351	4.6	2.8-7.3	0.011
MACE	359	24	12.7-40.6	297	23.2	13.1-37.6	0.933
MACE or mortality	2,440	18.5	10.5-30.4	2,237	9.6	3.5-23.4	0.231
MACE or mortality at follow-up	614	24.3	15.6-35.8	463	17.2	9.0-30.1	0.349

Abbreviation as in Table 1.

**Table 4:** Random-Effects Model Comparison of Present Postpartum and C-Section Prevalence in Cases

	Black PPCM			White PPCM			*P* Value
	n	%	95% CI	n	%	95% CI	
Present postpartum	389	84.3	76.3-90.6	297	84.1	70.2-92.6	0.9881
C-section	759	53.4	43.5-63.0	976	61.4	53.3-68.9	0.2161

Abbreviation as in Table 1.

**Figure 5:**
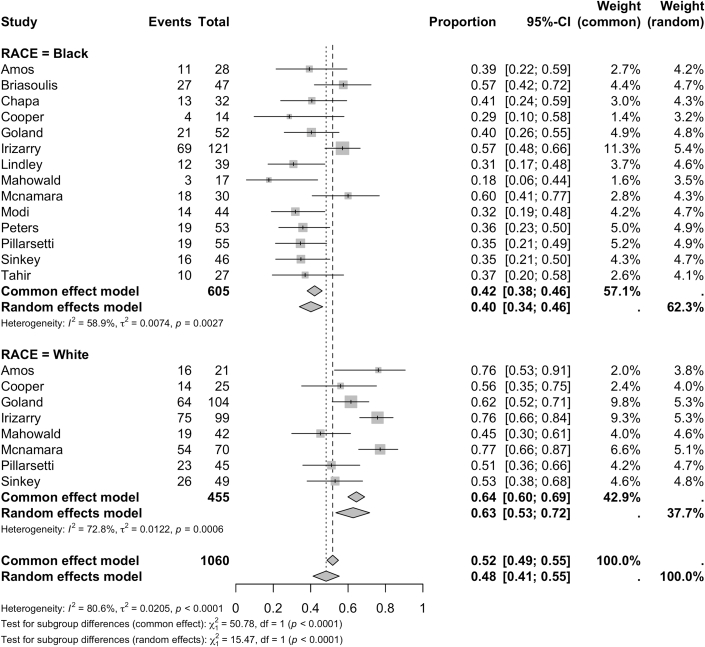
**Recovery of Ejection Fraction in Black and White Peripartum Cardiomyopathy Cases** Forest plot of pooled analysis of ejection fraction recovery in Black vs White women with peripartum cardiomyopathy.^47^, ^48^, ^49^, ^50^, ^51^, ^52^, ^53^, ^54^, ^55^, ^56^, ^57^

**Figure 6:**
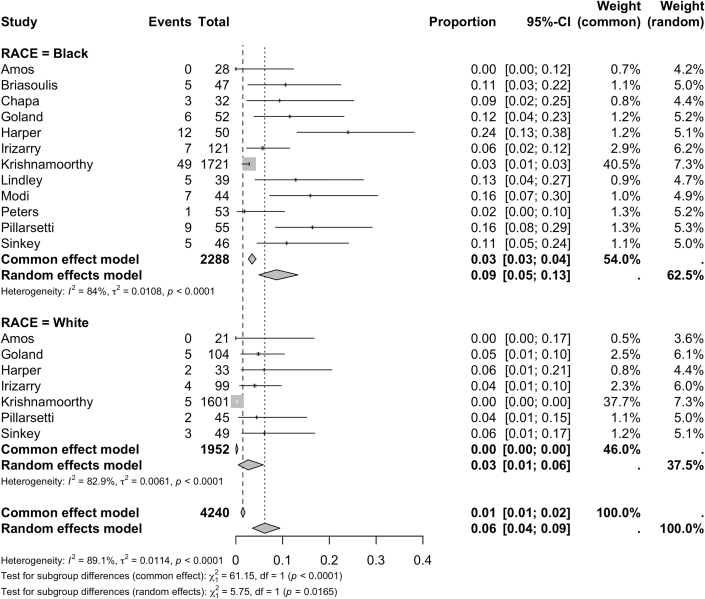
**Overall Mortality Rates in Black and White Peripartum Cardiomyopathy Cases** Forest plot of pooled analysis of death rates during acute hospitalization and follow-up periods in Black vs White women with peripartum cardiomyopathy.^47^, ^48^, ^49^, ^50^, ^51^, ^52^, ^53^, ^54^, ^55^, ^56^, ^57^

**Figure 7:**
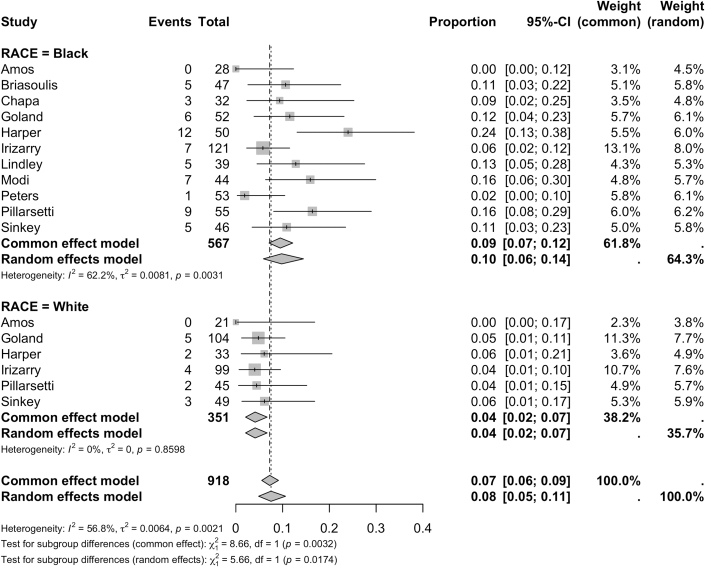
**Mortality Rates at Follow-up in Black and White Peripartum Cardiomyopathy Cases** Forest plot of pooled analysis of mortality rates recorded by studies with follow-up in Black vs White women with peripartum cardiomyopathy.^47^, ^48^, ^49^, ^50^, ^51^, ^52^, ^53^, ^54^, ^55^, ^56^, ^57^

When evaluated as a binary outcome, recovery was defined by EF >50% by 11 studies and >55% by 2 studies. Recovery was most often assessed between 6 and 12 months. Recovery rates differed between groups with 63% (95% CI: 54%-71%) of White women experiencing recovery compared to 40% (95% CI: 34%-46%) of Black women (*P* < 0.0001) (Figure 5). Black women demonstrated higher rates of mortality (8%, [95% CI: 5%-13%]) compared with White women (2%, [95% CI: 1%-6%], *P* = 0.013) (Figure 6). Black women experienced significantly higher rates of mortality at follow-up (10.3% vs 4.6% in White women, *P* = 0.011) and higher rates of in-hospital mortality although this difference was not significant (2.9% vs 1.4%; *P* = 0.540) (Figure 7). Both groups experienced similar rates of major cardiovascular outcomes (24% [95% CI: 13%-41%] in Black women vs 23 [95% CI: 13%-38%] in White women; *P* = 0.933) (Table 3).

We investigated differences in medication utilization between races to further understand differences in outcomes (Table 5). No significant difference was found between use of beta-blockers, angiotensin-converting enzyme inhibitors/angiotensin receptor blockers, aldosterone receptor antagonists, diuretics, digoxin, or calcium-channel blockers. A significant difference was found in utilization of nitrates, which was higher in Black cases (*P* = 0.022).

**Table 5:** Random-Effects Model Comparison of Present Postpartum and C-Section Prevalence in Cases

	Race	n	%	95% CI	*P* Value
Beta-blockers	Black	382	82.0%	67.2-91.0	0.725
Beta-blockers	White	263	85.1%	67.6-94.0	0.725
Angiotensin-converting enzyme inhibitors/angiotensin receptor blockers	Black	382	79.6%	73.5-84.6	0.289
Angiotensin-converting enzyme inhibitors/angiotensin receptor blockers	White	263	85.4%	75.0-91.9	0.289
Nitrates	Black	93	19.8%	12.9-29.1	0.022
Mineralocorticoid receptor antagonists	Black	132	29.0%	15.7-47.2	0.811
Digoxin	Black	176	31.3%	15.1-53.6	0.69
Diuretics	Black	129	79.1%	64.8-88.6	0.141
Anticoagulants	Black	83	36.1%	26.6-47.0	NA
Calcium channel blockers	Black	85	11.8%	6.4-20.5	0.296

## Discussion

Prior observational studies have reported disparities in PPCM between developed and developing countries, and between racial groups across countries.^21^^,^^22^ Quantitative and qualitative analyses in the form of a systematic review and meta-analysis have not been published addressing disparities in risk factors and outcomes for Black women with PPCM specifically in the United States. This study addresses this gap by analyzing available data to enhance understanding of risk factors, morbidity, and mortality associated with PPCM among Black and White women in the United States (Central Illustration).

**Central Illustration:**
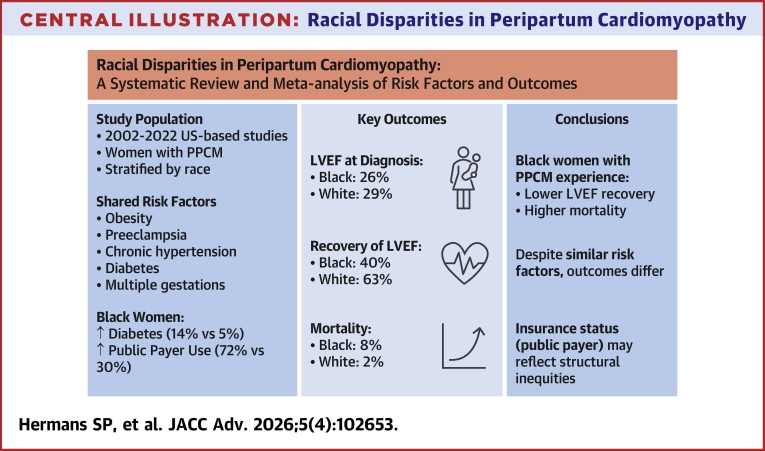
**Racial Disparities in Peripartum Cardiomyopathy** Shared risk factors illustrate risk factors that were significantly different in both Black and White cases compared to controls. At presentation, mean left ventricular ejection fraction was 26% in Black cases and 29% in White cases. Compared to White cases, Black cases had higher prevalence of diabetes and utilization of public payer, with no significant differences in preeclampsia, postpartum presentation, or other proposed contributors to disease pathophysiology or outcomes. Black women with peripartum cardiomyopathy experienced lower recovery and higher mortality, despite modest racial differences in previously identified risk factors. LVEF = left ventricular ejection fraction; other abbreviation as in Figure 2.

### Racial differences in risk factors

Established risk factors for PPCM include preeclampsia, hypertensive disorders of pregnancy, multiple gestation, and advanced maternal age.^23^, ^24^, ^25^, ^26^, ^27^ Others including obesity, diabetes, and tobacco use have more variable association across research. We found differences in prevalence for all investigated risk factors between cases and controls except age >30 years, payer status, and gestational hypertension, emphasizing the association of hypertensive and metabolic conditions with the development of PPCM.^14^^,^^27^

Prior research examining racial differences in established PPCM risk factors among general obstetric populations provides context in interpreting our study’s findings. Black American women have been reported to exhibit higher prevalence of PPCM risk factors compared to White women, which has been hypothesized to contribute to the increased incidence and mortality observed. Fingar et al reported an incidence of preeclampsia in 69.8 per 1,000 Black deliveries vs 43.3 per 1,000 White deliveries.^28^ Creanga et al and Centers for Disease Control and Prevention data reported a higher prevalence of obesity in Black pregnancies compared to non-Hispanic White pregnancies (39% vs 26%).^29^ Shah et al reported similar rates of gestational diabetes between groups (55.7 per 1,000 non-Hispanic Black births and 57.7 non-Hispanic White); however, disparate rates of pregestational diabetes (14.2 per 1,000 non-Hispanic Black births and 7.9 per 1,000 non-Hispanic Whites).^30^

According to Kao et al, Black controls were 4.5x more likely to utilize a public payer compared to White controls (59% vs 24%).^9^ These differences in payers may signify variance in health care access and demographics that may contribute to PPCM risk. While baseline prevalence of risk factors was higher among Black women, logistic regression in our study demonstrated that certain risk factors (obesity, preeclampsia, gestational hypertension, multiple gestations) had a stronger association with PPCM risk for White women vs Black women, and diabetes had a stronger association with PPCM risk in Black women. These differences may have implications with regard to risk assessment, monitoring, and management.

We hypothesized that differences in risk factor distribution between cases might explain observed disparities in outcomes^11^^,^^13^^,^^31^ (Figure 3). Consistent with prior research, White cases were 1.6x more likely to be over 30 years old compared to Black cases (*P* < 0.001). In our study, there was no significant difference in obesity; however, Black cases were more likely to have diabetes (14% vs 5%; *P* = 0.027). Black cases showed a modest increase in prevalence of chronic hypertension but it was not clinically significant. Furthermore, Black cases did not demonstrate higher rates of preeclampsia, tobacco use, or multiple gestations when compared with White cases.^9^^,^^11^^,^^32^^,^^33^ Overall, although diabetes prevalence differed between groups, there were no significant differences in other proposed cardiovascular and metabolic risk factors between races, which was an interesting discrepancy. Prior univariate and multivariate analyses investigating the association between diabetes and PPCM outcomes have been inconsistent and have failed to identify diabetes as a clear prognostic indicator in PPCM in the absence of other cardiometabolic risk factors.^34^, ^35^, ^36^ Further research is needed to understand how differences in the prevalence of diabetes in isolation of other risk factors may impact both short- and long-term outcomes such as death and recovery.

While differences between cases and controls highlight how defined risk factors may influence the development of PPCM, our findings do not support the hypothesis that differences in cardiovascular and metabolic risk factor profiles between Black and White cases lead to pathological differences in disease and differences in outcomes.

Sociodemographic factors emerged as a significant difference between groups, with 73% of Black cases utilizing a public payer compared to 30% of White cases. This disparity exemplifies unaccounted for sociodemographic factors and distribution of health care. Disparities in insurance may contribute to differing outcomes due to barriers to early diagnosis, access to care, quality of care and interventions, and long-term follow-up.^15^^,^^37^

### Racial differences in outcomes

Prior research has reported that Black cases are more likely to present postpartum and with lower EF, which has been hypothesized to contribute to worse outcomes.^11^^,^^38^ We found no difference in the percentage of Black cases presenting postpartum (84% [95% CI: 76%-90%]) compared with White cases. (84% [95% CI: 70%-92%]) (Table 4).

In our study, Black women presented with marginally lower baseline LVEF compared to White women (26% vs 30%) and were less likely to experience recovery (40% vs 63%). Of the 11 studies in our review that documented EF, 5 included Black cohorts, while 6 reported race-stratified cohorts. McNamara et al was the only study that found a significant difference in baseline EF between Black and White women (31% vs 36%).^39^ All other studies reported a nonsignificant difference in baseline EF with most reporting mean EF between 25% and 30%. While Black cases were noted to have a marginally lower EF at presentation, due to variability in imaging acquisition, operator skill, and subjective interpretation, the clinical significance of this small difference is uncertain, and it likely does not explain observed differences in recovery, given the influence of one study on the analysis results. Furthermore, the magnitude of difference in mean baseline EF (5% difference in EF) is much smaller than the magnitude of difference in mean recovery EF (23% difference in EF). The difference in baseline EF is within the expected margin of measurement error, while the difference in recovery EF is not.^40^

Recovery was assessed by 14 studies. All studies except one reported a significant difference in recovery. Our analysis found a significant disparity in recovery rates, aligned with prior reviews.^14^ While studies had widely variable rates of follow-up (6 months-76 months), prior research has suggested that LVEF usually improves with goal-directed medical therapy at 2 to 12 months.^14^^,^^38^

Black and White cases experienced similar rates of major cardiovascular outcomes (24% [95% CI: 13%-41%] in Black women vs 23% [95% CI: 13%-38%] in White women; *P* = 0.933) (Table 3). MACE was most often a composite endpoint, and limited data were available regarding specific event data such as implantable cardioverter-defibrillator (ICD), left ventricular assist device, and heart transplant, so additional pooled analyses were not conducted. Notably, of the 2 included studies investigating these outcomes, Sinkey et al found a significant difference in rates of ICD placement (B 33% vs W 14%, *P* = 0.03), but no significant difference in rates of left ventricular assist device (B 11% vs W 2%, *P* = 0.1), or heart transplant (B 2% vs W 0%, *P* = 0.23).^31^ Pillarisetti et al found no significant difference in ICD implantation or discharges between groups (B 14% vs W 11%; *P* = 0.6).^41^ Further investigation is needed to understand if differences in these interventions and access to care may contribute to differences in outcomes.

We also investigated whether differences in medications could explain observed outcomes. Prior research has failed to demonstrate consistent differences in utilization of heart failure medications. We found limited differences in prescribed medications, aside from significant difference in the use of nitrates. While limited by small sample size, our results suggest that differences in medical therapies cannot adequately explain differences in outcomes.

In our study, Black women experienced higher rates of mortality compared to White women (8% [95% CI: 5%-13%] vs 2% [95% CI: 1%-6%]; *P* = 0.013). While heterogeneity was substantial between studies due to changes in follow-up time, when mortality was limited to studies with follow-up, the disparities persisted (10.3% in Black patients vs 4.6% in White women, *P* = 0.011), Previous studies have suggested that mortality rates in Black women with PPCM resemble those seen in developing nations rather than those of their White U.S. counterparts.^11^^,^^13^ Kerpen et al found a PPCM related mortality rate in developing countries of 14%, compared to 4% in advanced countries; notably, no difference was found in the prevalence of risk factors between advanced and developing countries.^22^ Authors concluded that PPCM related mortality was higher in Black women internationally. Worse outcomes in developing countries have been linked to disparities in healthcare access, quality of care, and differences in education.^22^

The mortality rate observed in our study was 4x higher in Black cases compared to White cases. This finding is congruent with other discouraging outcomes data for pregnant Black women in the United States. Wang et al (2020) found that minority women had 1.2-5x higher risk of severe maternal morbidity compared to White women.^42^ Johnson et al demonstrated a maternal mortality rate 3x higher in Black women compared to White women (69.9 vs 26.6 per 100,000 live births).^43^ A thorough discussion of the etiologies of these disparities is outside the scope of our work, though we found significant differences in age, diabetes diagnoses, and payer status. These differences most likely contribute to but incompletely explain the mortality gap between Black and White women. We hypothesize that systemic factors such as access to care and structural racism may contribute to the observed disparities in outcomes. Further investigation to evaluate clinical risk differences and systemic factors, and the nuanced relationships between them, will be crucial to addressing disparities with policy interventions to help reduce maternal mortality rates. Access to care may be improved by increased utilization of telemedicine, home intervention programs, or care at multidisciplinary clinics. Efforts to address systemic factors may include provider-level interventions such as education to improve awareness of implicit biases and efforts towards increased workforce diversity. Lastly, interventions to address systemic disparities may include changes to Medicaid eligibility and reimbursement, increased funding for community maternal health programs, improved data collection on maternal health disparities, and increased access to basic needs such as transportation, housing, and food.^44^^,^^45^

### Study limitations

Our study had several limitations. High heterogeneity was a limitation, and significant heterogeneity was observed in recovery (I^2^ = 80.6%), MACE (I^2^ = 87.1), and mortality (I^2^ = 89.1%). Less heterogeneity was observed for mortality at follow-up (I^2^ = 56.8) and MACE or Mortality at Follow-up (I^2^ = 56.8). Definitions of MACE, EF recovery (55% vs 50%), and inclusion of timing of diabetes diagnosis (overall, pregestational/chronic, gestational) varied, as did follow-up time and time to repeat echocardiogram. Reduction in heterogeneity with restriction to studies including follow-up demonstrate the significance of follow-up time in between study variability. The heterogeneity observed in this study likely reflects important differences across studies in addition to follow-up time, such as study design, patient characteristics, and outcomes. Due to heterogeneity, risk factors may have been underreported or overreported and the generalizability in the pooled estimates found may be limited. A sensitivity analysis was not feasible due to limited number of studies available for risk factors and outcomes. Since all studies took place in academic centers, there was likely institutional bias, and higher morbidity and mortality compared to general population. Reliance on medical record data resulted in limitations in understanding confounding factors including education, social support, and access to care. Our study was limited by small sample sizes in subgroup analysis.

Furthermore, as discussed by Wang et al there is increasing complexity in measuring the social construct of race through administrative and self-reported data.^42^ Scholars have proposed that race is not an isolated factor but a marker of risk for racism-related exposures.^42^^,^^46^ Other limitations include selection bias, inconsistent primary outcomes, standardization of echocardiogram, small samples, retrospective design, single center limitations, coding misclassification, and incomplete data capture. Lastly, while this study investigated differences between race matched cases, data on race matched controls was limited so meta-analysis was not conducted for controls. This limited our ability to fully account for differences in baseline risk factor prevalence among controls and their influence on development of disease.

## Conclusions

To the best of our knowledge, this is the first systematic review and meta-analysis to quantify and analyze racial disparities in risk factors and outcomes related to PPCM in U.S. women. This study confirms the association between cardiovascular and metabolic risk factors and the development of PPCM across racial groups in the United States, and highlights significant racial disparities in PPCM related morbidity and mortality. Modest but significant differences in prevalence of several proposed risk factors were observed between Black and White cases. One large observed difference between Black cases and White cases was payer status. These findings suggest that healthcare disparities, social determinants of health, and access to care may influence outcomes. However, the data available provided limited adjustment for sociodemographic factors. Future research should investigate differences in treatment, follow-up care, and unaccounted social determinants of health to better understand and address disparities in PPCM outcomes.

Perspectives **COMPETENCY IN MEDICAL KNOWLEDGE AND PROFESSIONALISM:** Black women living in the United States with PPCM experience significantly worse outcomes compared to White women, despite minimal identified differences in risk factors and treatment. (Medical Knowledge, Professionalism).

**TRANSLATIONAL OUTLOOK:** Studies further investigating differences in social determinants of health may help to clarify racial disparities in PPCM outcomes in the United States. This future work is crucial to developing effective individual and systemic interventions.

## Funding support and author disclosures

The authors received statistical support funded by the Department of Internal Medicine at the Ohio State University College of Medicine. The authors have reported that they have no relationships relevant to the contents of this paper to disclose.
