# Supplementary material for: Racial Disparities in U.S. Peripartum Cardiomyopathy: Systematic Review and Meta-Analysis of Risk Factors and Outcomes
Source: JACC Adv. 2026 Mar 16;5(4):102653. doi: 10.1016/j.jacadv.2026.102653 (PMC13011245; doi:10.1016/j.jacadv.2026.102653)
Supplement: Supplemental Material [file mmc1.docx]

**Supplementary Appendix A: Study Protocol**

Disparities in risk factors and outcomes between Black and White women with peripartum cardiomyopathy in the United States: Protocol for a systematic review and meta-analysis

Sarah Perou Hermans, MD, MPH,^1^ Alexander N. Arreguin, MD,^1^ Lauren J. Hassen, MD, MPH^2^

1. Department of Internal Medicine, The Ohio State University Wexner Medical Center, Columbus, OH, USA, 43210
2. Department of Internal Medicine*,* Division of Cardiovascular Medicine*,* The Ohio State University Wexner Medical Center, Columbus, OH, USA, 43210

**STUDY BACKGROUND, RATIONALE, AND OBJECTIVES**

***Background***

Peripartum cardiomyopathy (PPCM) is a common cause of heart failure in pregnancy and a significant contributor to maternal morbidity and mortality. It is characterized by the development of heart failure in women with no prior cardiovascular disease and occurs late in pregnancy or within five months postpartum.^1^ The reported incidence of PPCM varies across different regions and ethnicities, with published rates ranging from 1 in 100 to 1 in 15,500 live births.^2^ Reported mortality rates range between 4–14% within the first 12 months postpartum.^3^ Women of African ancestry have been found to be disproportionately affected by PPCM and experience worse outcomes compared to other racial groups.^4^

Some studies have demonstrated race, specifically African-American ethnicity, to be the most important predictor of PPCM development.^4^ The incidence of peripartum cardiomyopathy in Black American women has been reported to be 2.7-4.6 times higher compared to White Americans.^5-10^ Black patients are more likely to present postpartum and with more severe symptoms of heart failure.^11,12^ Among Black American women with PPCM who recovered, time to recovery was twice as long as those of other racial groups.^12^ Furthermore, Black American women with PPCM have higher mortality rates and decreased left ventricular recovery rates compared to white patients, with some studies suggesting these outcomes are more comparable to women in Haitian and South African populations.^11,13^

Differences in epidemiology and outcomes between PPCM patients of African descent and those of other ethnicities have been attributed to various factors, including genetic variants, autoimmunity, angiogenic imbalance, pre-existing health conditions, variations in disease pathophysiology, and socioeconomic disparities including nutrition, limited access to care, and education.^14^ Contributing factors may also include a higher incidence of risk factors for PPCM such as hypertensive disorders of pregnancy, multiple gestations, tobacco use, and diabetes.^9, 14^ Published data identifying differences in the prevalence of these risk factors between Black and White women diagnosed with PPCM have been inconsistent. Several studies have found the increased risk in Black American women is independent from sociodemographic factors such as poverty, smoking, and lower education level, and independent from differences in medical treatment.^4, 11, 15^ This study aims to extend previous research by further quantifying differences in known risk factors between Black and White populations to gain further understanding behind the increased incidence, morbidity, and mortality in this population.

***Rationale***

Previous research has demonstrated ethnicity and African descent to be a significant risk factor in the development of PPCM. Primary observational studies have highlighted disparities in PPCM incidence, morbidity, and mortality between Black and White women from the United States. To our knowledge, no systematic review and meta-analysis has been conducted to quantify and analyze disparities in risk factors and outcomes related to PPCM in Black women from the United States.

***Study Objectives***

Through systematic review and meta-analytic synthesis of all current available relevant research literature this study aims to:

1. Quantify differences in risk factors associated with the development of PPCM in Black women and White women in the United States.
2. Evaluate differences in PPCM-related outcomes including a primary outcome of mortality and secondary outcomes of recovery, and adverse events between Black and White women in the United States.

**LITERATURE AND DATA SEARCH AND REVIEW**

This systematic review and meta-analysis follows the guidelines set by Preferred Reporting Items for Systematic Reviews and Meta-Analysis (PRISMA).^16^ This study was registered with PROSPERO (CRD42023439228). Experts in library science were consulted. The following PICOS framework was applied: population (Black women diagnosed with PPCM in the United States), intervention (none), comparison (White US women with PPCM), outcomes (primary – mortality, secondary – recovery, adverse events, morbidity), and study design (cohort, case control, cross sectional studies). A protocol and checklist of inclusion criteria were developed to guide the reviewers through the screening process.

***Electronic search and search strategy***

Bibliographic databases utilized included Embase (Elsevier, Amsterdam, Netherlands), MEDLINE (PubMed; National Library of Medicine, NIH, Bethesda, MD, USA), Web of Science (Clarivate Analytics, Philadelphia, PA, USA), and Google Scholar (Google, Mountain View, CA, USA). Searches were limited to English language articles published after January 01, 2002. Search terms were used to exclude case reports, letters, commentaries, case series, editorials. The following PubMed search strategy was employed and altered to fit other databases (("Peripartum Period"[Mesh] OR "Peripartum Period" OR "Peripartum" OR "Peripartum Women" OR "Peripartum Woman") AND ("Cardiomyopathies"[Mesh] OR "Cardiomyopathies" OR "Cardiomyopathy")) OR (("Peripartum Cardiomyopathy" OR "Peri-partum Cardiomyopathy" OR ‘‘Peripartum-Associated Cardiomyopathy’’ OR "Postpartum Cardiomyopathy" OR "Post-partum Cardiomyopathy" OR "PPCM")).

***Search of other resources***

References and citations of all qualifying articles were reviewed to identify further studies of interest. Previously conducted meta-analysis on peripartum cardiomyopathy were reviewed to ensure all relevant studies have been included. A gray literature search was conducted through Google Scholar which includes books, dissertations, conference presentations, and government reports. An additional search was conducted through the Networked Digital Library of Theses and Dissertations.^17^

***Literature screening***

Articles retrieved from the literature search were imported into Covidence and duplicates were removed. Titles and abstracts were then screened for relevance by two reviewers (SH and AA). References were selected based on the selection criteria applied to both title and abstract. References with only titles and without abstracts were carried through to next phase. Exclusion criteria during this stage is detailed in the following section. References screened by the reviewers were then compared and the full text of overlapping references was obtained. Non-overlapping references were reviewed again by both reviewers prior to obtaining full text. Once full texts were obtained, two independent reviewers screened all articles. Reasons for exclusion were recorded. For studies with data overlap, the results were analyzed as a single sample OR the study with the largest sample size was included.

**ELIGIBILITY CRITERIA**

***Inclusion and exclusion criteria***

During screening of abstracts and titles, as detailed in the literature and data search section, studies were excluded if they did not meet the following criteria:

1) no mention of peripartum cardiomyopathy

2) not in the English language

3) not a United States patient population

4) not a primary research article

5) publication date prior to 2002

6) sample size <20

During review of full text articles, the following exclusion criteria were applied and reasons for exclusion were recorded and quantified per PRISMA guidelines:

1) sample not race-stratified or not predominately Black (>74%)

2) sample size <20

3) inadequate data

4) incorrect study design

5) incorrect patient population

6) incorrect outcomes or differing outcomes recorded

7) race was analyzed as a co-variate instead of as an exposure.

***Study population***

Participants in this study include women with a diagnosis of peripartum cardiomyopathy meeting definitions set by the NHLBI/ORD Workshop definition or if categorized by ICD-9-CM 674.50 to 55, or ICD-10-CM O90.3. The NHLBI/ORD workshop defines PPCM as 1) development of heart failure in the last month of pregnancy or within 5 months after delivery 2) absence of another identifiable cause of HF 3) absence of recognizable heart disease before the last month of pregnancy and 4) LV systolic dysfunction demonstrated by classic echocardiographic criteria, such as depressed EF <45%.^1^ The population of interest includes women diagnosed with PPCM in the United States, with a specific focus on Black and White women. Only studies with a sample consisting of women diagnosed with PPCM in a US medical institution will be included. Participants with pre-existing heart disease or who were diagnosed with peripartum cardiomyopathy in prior pregnancies will not be included.

***Risk factor and outcome measures***

The aim of our study is to describe and quantify differences in risk factors and outcomes between the two groups. We included all studies that report predefined risk factors for peripartum cardiomyopathy stratified by race or belonging to a majority Black patient population AND/OR studies that include outcomes such as mortality, morbidity, and recovery stratified by race or belonging to a majority Black population.

***Types of studies***

Included studies in this meta-analysis were published within 20 years of study initiation to increase external validity given medical advances. Only studies consisting of women with peripartum cardiomyopathy who were diagnosed in a United States medical institution were included. Studies included in this meta-analysis were primary observational studies such as cross-sectional, case-control, and cohort studies. Meta-analyses, case reports, commentaries, and editorials were excluded, although case series, letters, and the control arm of randomized clinical trials were reviewed for potential inclusion.

**DATA EXTRACTION AND MANAGEMENT**

Data extraction was conducted by two reviewers (AA and SPH) after final identification of qualifying full-text studies. Covidence was used to assist with data extraction. A secure Excel document will be used to record general characteristics of study, investigator name, title and journal of publication, years of study, sample source, study type, study location, definition of diagnosis of PPCM, sample size stratified by race, as well as average follow-up period. The following predefined risk factors of peripartum cardiomyopathy will be recorded when reported by qualifying studies: age >30 years old, obesity, hypertensive disorders of pregnancy (preeclampsia, eclampsia, gestational hypertension), chronic hypertension, diabetes, gestational diabetes, multiple gestations, multiparity, tobacco or substance use, and payor. Outcome measures recorded included a primary outcome of mortality and secondary outcomes of recovery of ejection fraction at follow-up, morbidity or maternal major cardiovascular event, or c-section. Maternal major cardiovascular event will be defined as a composite of pre-defined outcomes related to PPCM, and differing definitions of MACE will be recordedd.^18^

**ASSESSMENT OF METHODOLOGICAL QUALITY**

Reviewers SPH and AA assessed all included studies for bias by grading each study independently through use of Newcastle–Ottawa Scale for observational studies.^19^

**REFERENCES**

1. Pearson GD, Veille JC, Rahimtoola S, et al. Peripartum Cardiomyopathy. National Heart, Lung, and Blood Institute and Office of Rare Diseases (National Institutes of Health) Workshop Recommendations and Review. *JAMA*. 2000;283(9):1183-1188. doi:https://doi.org/10.1001/jama.283.9.1183
2. Isogai T, Kamiya CA. Worldwide Incidence of Peripartum Cardiomyopathy and Overall Maternal Mortality. *International Heart Journal*. 2019;60(3):503-511. doi:https://doi.org/10.1536/ihj.18-729
3. Sliwa K, Petrie MC, Hilfiker-Kleiner D, et al. Long-term prognosis, subsequent pregnancy, contraception and overall management of peripartum cardiomyopathy: practical guidance paper from the Heart Failure Association of the European Society of Cardiology Study Group on Peripartum Cardiomyopathy. *European Journal of Heart Failure*. 2018;20(6):951-962. doi:https://doi.org/10.1002/ejhf.1178
4. Gentry MB, Dias JK, Luis A, Patel R, Thornton J, Reed GL. African-American Women Have a Higher Risk for Developing Peripartum Cardiomyopathy. *Journal of the American College of Cardiology*. 2010;55(7):654-659. doi:https://doi.org/10.1016/j.jacc.2009.09.043
5. Brar SS, Khan SS, Sandhu GK, et al. Incidence, Mortality, and Racial Differences in Peripartum Cardiomyopathy. *The American Journal of Cardiology*. 2007;100(2):302-304. doi:https://doi.org/10.1016/j.amjcard.2007.02.092
6. Gunderson EP, Croen LA, Chiang V, Yoshida CK, Walton D, Go AS. Epidemiology of peripartum cardiomyopathy: incidence, predictors, and outcomes*. Obstet Gynecol* 2011;118:583—91.
7. Ijaz SH, Jamal S, Mannan A, et al. Trends in Characteristics and Outcomes of Peripartum Cardiomyopathy Hospitalizations in the United States Between 2004 and 2018. *The American Journal of Cardiology*. 2022;168:142-150. doi:https://doi.org/10.1016/j.amjcard.2021.12.034
8. Kolte D, Khera S, Aronow WS, et al. Temporal Trends in Incidence and Outcomes of Peripartum Cardiomyopathy in the United States: A Nationwide Population‐Based Study. *Journal of the American Heart Association*. 2014;3(3). doi:https://doi.org/10.1161/jaha.114.001056
9. Kao DP, Hsich E, Lindenfeld J. Characteristics, Adverse Events, and Racial Differences Among Delivering Mothers With Peripartum Cardiomyopathy. *JACC: Heart Failure*. 2013;1(5):409-416. doi:https://doi.org/10.1016/j.jchf.2013.04.011
10. Phan D, Duan L, Ng A, Albert Yuh-Jer Shen, Lee MS. Characteristics and outcomes of pregnant women with cardiomyopathy stratified by etiologies: A population-based study. *International Journal of Cardiology*. 2020;305:87-91. doi:https://doi.org/10.1016/j.ijcard.2019.12.027
11. Goland S, Modi K, Bitar F, et al. Clinical Profile and Predictors of Complications in Peripartum Cardiomyopathy. *Journal of Cardiac Failure*. 2009;15(8):645-650. doi:https://doi.org/10.1016/j.cardfail.2009.03.008
12. Irizarry OC, Levine LD, Lewey J, et al. Comparison of Clinical Characteristics and Outcomes of Peripartum Cardiomyopathy Between African American and Non–African American Women. *JAMA Cardiology*. 2017;2(11):1256. doi:https://doi.org/10.1001/jamacardio.2017.3574
13. Modi K, Illum S, Karim Jariatul, Caldito G, Reddy PK. Poor outcome of indigent patients with peripartum cardiomyopathy in the United States. *Am J Obstet Gynecol*. 2009;201(2):171.e1-171.e5. doi:https://doi.org/10.1016/j.ajog.2009.04.037
14. Elkayam U, Tummala PP, Rao K, et al. Maternal and Fetal Outcomes of Subsequent Pregnancies in Women with Peripartum Cardiomyopathy. *New England Journal of Medicine*. 2001;344(21):1567-1571. doi:https://doi.org/10.1056/nejm200105243442101
15. Ukah UV, Li X, Wei SQ, Healy-Profitós J, Dayan N, Auger N. Black-White disparity in severe cardiovascular maternal morbidity: A systematic review and meta-analysis. *American Heart Journal*. 2022;254:35-47. doi:https://doi.org/10.1016/j.ahj.2022.07.009
16. Page M, Joanne M, Patrick B, et al. The PRISMA 2020 statement: an updated guideline for reporting systematic reviews. *BMJ*. 2021;372(71). doi:https://doi.org/10.1136/bmj.n71
17. The Journal of Electronic Theses and Dissertations . Networked Digital Library of Theses and Dissertations. search.ndltd.org. Accessed June 24, 2023. <http://search.ndltd.org>
18. Owens A, Yang J, Nie L, Lima F, Avila C, Stergiopoulos K. Neonatal and Maternal Outcomes in Pregnant Women With Cardiac Disease. *Journal of the American Heart Association*. 2018;7(21). doi:https://doi.org/10.1161/jaha.118.009395
19. Wells G, Shea B, O’Connell D, et al. The Newcastle-Ottawa Scale (NOS) for assessing the quality of nonrandomised studies in meta-analyses. www.ohri.ca. Published 2021. Accessed June 26, 2023. https://www.ohri.ca/programs/clinical_epidemiology/oxford.asp

**Supplementary Appendix B: Qualitative Analysis**

**Cohort studies**

| Study Author | Total stars | Case definition | Representativeness of the exposed cohort | Selection of the non exposed cohort | Selection of comparative group | Ascertainment of exposure | Outcome of interest not present at start | Assessment of outcome |
| --- | --- | --- | --- | --- | --- | --- | --- | --- |
|  |  |  |  |  |  |  |  |  |
| Afana | 6 of 9 | B | C | A | NA | A | A | B |
| Amos | 5 of 9 | A | B | NA | NA | A | A | A |
| Briasoulis | 9 of 9 | A | B | A | NA | A | A | A |
| Chapa | 6 of 9 | A | B | NA | NA | A | A | A |
| Cooper | 6 of 9 | A | A | NA | A | A | A | A |
| Gambahaya | 7 of 9 | B | B | A | NA | A | A | B |
| Goland | 7 of 9 | A | A | A | NA | A | A | A |
| Harper | 8 of 9 | A | A | A | NA | A | A | B |
| Irizzary | 5 of 9 | A | A | A | NA | A | A | A |
| Kao | 7 of 9 | B | C | A | NA | A | A | A |
| Krishnamoorthy | 6 of 9 | B | A | NA | A | A | A | B |
| Lewey | 7 of 9 | A | A | A | NA | A | A | A |
| Lindley | 6 of 9 | B | A | NA | A | A | A | B |
| Mahowald | 6 of 9 | A | A | NA | A | A | A | B |
| Mcnamara | 8 of 9 | A | A | NA | A | A | A | A |
| Modi | 7 of 9 | A | B | NA | NA | A | A | A |
| Peters | 6 of 9 | A | A | NA | A | A | A | A |
| Pillarsetti | 9 of 9 | A | A | NA | A | A | A | A |
| Sinkey | 6 of 9 | A | A | NA | A | A | A | A |
| Tahir | 9 of 9 | A | A | NA | A | A | A | A |

Case/ Control Studies

|  |  | Case definition | Representativeness of the cases | selection of controls | definition of controls | Ascertainment of exposure | Assessment of outcome | Ascertainment of exposure |
| --- | --- | --- | --- | --- | --- | --- | --- | --- |
| Gentry | 9 of 9 | A | A | A | A | A | B | A |

**Qualitative Analysis Continued**

Afana

- Comparability/ adjusts for - Age, race/ ethnicity, comorbidities
- end outcome reported - Combined end point was defined as death, cardiac arrest, cardiogenic shock, or use of extracorporeal membrane oxygenation or intra-aortic balloon pump.
- Follow-up time / variation – no follow-up
- Variation in follow-up period – none
- Follow-up long enough to assess outcome: No
- Excluded populations - Any case with ICD-9-CM codes for other cardiomyopathies and pre-existing cardiac dysfunction. ICD9-CM codes for other ill-defined/unspecified heart disease, human immunodeficiency virus, and male sex
- Bias reported - Potential insufficient coding/ coding error. Limited to PPCM w/ delivery during hospitalization when they often px post partum. Underestimates long term adverse outcomes. Limited to delivery. Possible duplicate patients with different delivery times. Limited size of comparison group may introduce unmeasured confounding variables.

Amos

- Comparability/ adjusts for - NA
- End outcome reported - Recovery, time to recovery, major adverse outcomes (Death, transplant, major adverse outcome, rehospitalization)
- Variation in follow-up - 43 m (range 0.2 to 90 months) BUT repeat echo for recovery at 2m
- Adequacy of follow-up - 49 of 55
- Follow-up long enough to assess outcome: Yes
- Excluded populations - Exclusion criteria included a significant comorbid illnesses (including HIV, cancer, and severe lung disease) and history of chemotherapy use.
- Bias reported - Referral institution/tertiary care center, patients may have worse prognosis. Patients may have died prior to transfer. Small sample size. Lack of control group. Limited generalizability

Briasoulis

- Comparability/ adjusts for - Clinically significant factors (age, race, hypertension, diabetes, EF, and GLS)
- End outcome reported - Longitudinal systolic strain profiles and outcomes including all-cause mortality or the composite endpoint of all-cause mortality, rehospitalization, or no LVEF recovery
- Variation in follow-up: mean 12.5 months, range 1-60 months
- Adequacy of follow-up:47 of 47 clinically, 45/47 follow-up echo
- Follow-up long enough to assess outcome: Yes
- Excluded populations: Patients with preeclampsia and uncontrolled hypertension were excluded.
- Bias reported: Limited by small sample size, some issues with lack of follow-up echo data. No ability to verify diagnostic accuracy

Chapa

- Comparability/ adjusts for NA
- End outcome reported: Persistent cardiac dysfunction compared to EF at diagnosis
- Variation in follow-up period: Most w/ recovery had it at 3 month FU, and those with persistent dysfunction followed for 46 months
- Adequacy of follow-up: 32 of 35
- Follow-up long enough to assess outcome: Yes
- Excluded populations: Pre-existing disease, such as idiopathic dilated cardiomyopathy or concurrent cardiovascular risk factors, including hypertension and alcoholism, were the primary reasons for patient exclusion. OF note some other studies included pre-existing HTN.
- Bias reported : Limited by small sample size. Additionally, the cases occurred over a 13-year time period, during which significant advances in cardiac care have been made. study include 3 patients in our cohort whose timing of disease is outside the previously suggested time period for diagnosis.

Cooper

- Comparability/ adjusts for - None
- End outcome reported - Myocardial recovery. Change in LVEF from baseline to 6m via echo. The occurrence of hospitalization, cardiac transplantation, or death was also noted
- Variation in follow-up period - Follow up data is at 6m (recovery, EF). Average F/U otherwise 781 days +/- 516 days
- Adequacy of follow-up - 14 of 14 Black cases
- Follow-up long enough to assess outcome: Yes
- Excluded populations Patients with significant diabetes (re- quiring therapy with insulin or an oral agent for more than 1 year), and uncontrolled hypertension (diastolic greater than 95 mm Hg or systolic 160 mm Hg) were excluded. Subjects underwent angiography or non-invasive assessment to exclude coronary artery disease, and transthoracic echocardiogram to rule out significant valvular disease.
- Bias reported - Small sample size, ascertainment bias, center variability.

**Gambahaya**

- Comparability / adjusts for – CV risk factors (chronic hypertension, diabetes, and obesity) and socioeconomic factors (insurance status, hospital income, and residential income)
- End outcome reported – Adverse cardiovascular (CV) outcomes during delivery hospitalization
- Variation in follow-up period – None
- Adequacy of follow-up – No follow-up
- Follow-up long enough to assess outcome – No
- Excluded populations – Restricted to delivery hospitalizations; excluded post-discharge PPCM diagnoses and women outside the delivery setting
- Bias reported – Assessment limited to only immediate in-hospital outcomes. Bias risk in misclassification from ICD codes, lack of post-discharge data, and inability to adjust for all confounders

Goland

- Comparability / adjusts for – Multivariable model not fully adjusted for all confounders
- End outcome reported – LV recovery; number of major adverse events; death; combined rate of death and heart transplantation
- Variation in follow-up period – Mean follow-up 19 ± 14 months
- Adequacy of follow-up – 52 of 52
- Follow-up long enough to assess outcome – Yes
- Excluded populations – Women with pre-existing heart disease or LVEF >45%
- Bias reported – Retrospective nature; selection and reporting bias; referral bias

Harper

- Comparability / adjusts for – Age, race, insurance, delivery mode, and comorbidities
- End outcome reported – Case-fatality and complications during delivery hospitalization
- Variation in follow-up period – Followed for 7 years
- Adequacy of follow-up – 79 of 79
- Follow-up long enough to assess outcome – Yes
- Excluded populations – Women with recent viral-like illness, hypertrophic cardiomyopathy, or pulmonary edema with normal LV function
- Bias reported – Strict inclusion criteria; possibly sicker patient population; ICD-9 codes may not have captured all cases; 42 charts inaccessible; mortality coding may under- or over-estimate outcomes; no outpatient or postpartum follow-up

Irizzary

- Comparability / adjusts for – No multivariate adjustment
- End outcome reported – Cardiac recovery, time to recovery, cardiac transplant, persistent dysfunction
- Variation in follow-up period – None (only follow-up at 6 months)
- Adequacy of follow-up – 63 of 123
- Follow-up long enough to assess outcome – 6 months, somewhat
- Excluded populations – Pre-existing HF or cardiomyopathy, those not meeting PPCM diagnostic criteria
- Bias reported – Referral center; possible data misclassification; small sample size; single-center data

Kao

- Comparability / adjusts for – Demographics, comorbidities, race, presence of PPCM at delivery
- End outcome reported – The primary outcome was a composite of major adverse events (MAEs): death, cardiac arrest, heart transplantation, and/or mechanical circulatory support. Secondary outcomes included individual components of the primary endpoint, length of hospital stay, cesarean delivery, and stillbirth.
- Variation in follow-up period – None
- Adequacy of follow-up – No follow-up
- Follow-up long enough to assess outcome – No
- Excluded populations – PPCM patients hospitalized after delivery; excluded 1,459 records without delivery outcome
- Bias reported – No postpartum data; limited to delivery; possible duplicate patients

Krishnamoorthy

- Comparability / adjusts for – Demographics, cardiovascular and other risk factors
- End outcome reported – In-hospital mortality
- Variation in follow-up period – None
- Adequacy of follow-up – No follow-up
- Follow-up long enough to assess outcome – No
- Excluded populations – Non-delivery hospitalizations, incomplete data
- Bias reported – Diagnostic code misclassification; unclear active vs secondary diagnoses; no follow-up data; no control group

Lewey

- Comparability / adjusts for – Race, diagnosis timing, baseline EF
- End outcome reported – Full LV recovery (EF >50%); time to recovery; major adverse events (LVAD, transplant, death)
- Variation in follow-up period – 42.6 months (IQR 9.5–89.2)
- Adequacy of follow-up – 58 of 104
- Follow-up long enough to assess outcome – Yes
- Excluded populations – Congenital heart disease, valvular disease, cardiotoxic chemo, non-live births, inadequate EF data
- Bias reported – Retrospective cohort over 30 years; incomplete capture; variation in follow-up; referral bias; missing data; single-center

Lindley

- Comparability / adjusts for – No multivariate adjustment
- End outcome reported – One-year event-free survival; secondary: EF recovery
- Variation in follow-up period – not explicitly stated, did have 12m assessment, echo performed between 6 and 24 months after diagnosis.
- Adequacy of follow-up – 32 of 39
- Follow-up long enough to assess outcome – Yes
- Excluded populations – Initial echo not available; limited to delivery hospitalizations; excluded postpartum readmissions
- Bias reported – Small sample; retrospective; single tertiary center; coding misclassification; selection bias

Mahowald

- Comparability / adjusts for – No multivariate adjustment
- End outcome reported – All-cause mortality, MAE (death, transplant, LVAD, inotrope-dependence)
- Variation in follow-up period – 76 ± 7.8 months
- Adequacy of follow-up – 17 Black patients had EF follow-up (59 overall)
- Follow-up long enough to assess outcome – Yes
- Excluded populations – Other HF causes, chemo, myocarditis, drug-induced CM, insufficient info, lack of echo
- Bias reported – Single-center; retrospective; tertiary referral center; EF variability; no medication data

Mcnamara

- Comparability / adjusts for – Race, baseline EF
- End outcome reported – Survival free of death, transplant, LVAD; predictors: EF, LVEDD, race
- Variation in follow-up period – One year (6 & 12m)
- Adequacy of follow-up – 97% at 6m, 91% at 12m
- Follow-up long enough to assess outcome – Somewhat (12m)
- Excluded populations – Valvular/ischemic disease, septicemia, drug/alcohol abuse, chemo/radiation, prior cardiomyopathy
- Bias reported – Variation in the time post-partum to study entry, from the day of delivery to nearly 3months post-partum, sample size, selection bias

Modi

- Comparability / adjusts for – No matched controls; no multivariate adjustment
- End outcome reported – Mortality rate, LV recovery
- Variation in follow-up period – 24 (range, 0.1-264) months All patients with the diagnosis of PPCM had an echo-cardiographic assessment at diagnosis, 9
- months, and again at the last follow-up visit.
- Adequacy of follow-up – 40 of 44
- Follow-up long enough to assess outcome – Yes
- Excluded populations – Not specified
- Bias reported – Single-center; retrospective; no controls; small sample; uncontrolled confounding; limited analysis

Peters

- Comparability / adjusts for – EF <30%, LVEDD ≥60mm, MR severity, era (UNOS)
- End outcome reported – Composite of LVAD, transplant, death
- Variation in follow-up period – Median 3.6 years (1.4–7.3)
- Adequacy of follow-up – 53 of 53 ( 1 excluded in beginning )
- Follow-up long enough to assess outcome – Yes
- Excluded populations – Prior PPCM, age <18, insufficient follow-up
- Bias reported – ICD miscoding; referral bias; incomplete echo review; missing RV function; retrospective; limited diversity

Pillarsetti

- Comparability / adjusts for – Significant covariates
- End outcome reported – LVEF, ICD survival
- Variation in follow-up period – 35 ± 21 months
- Adequacy of follow-up – 55 Black patients (100 overall, 7 excluded)
- Follow-up long enough to assess outcome – Yes
- Excluded populations – Pre-existing cardiomyopathy, EF >45%, inadequate echo data, missing diagnostic codes
- Bias reported – Retrospective; exact cause of death not always known; medication dosing missing; difficult to assess ICD benefit

Sinkey

- Comparability / adjusts for – No multivariate adjustment
- End outcome reported – EF at diagnosis; recovery; persistent dysfunction; ECMO, HD, CRRT, LVAD, transplant, death
- Variation in follow-up period – 18 years (not explicitly described)
- Adequacy of follow-up – does not report LTFU but does report significant missing data. Initial black population was 46, It seems that 19 missing on follow-up EF for persistent dysfunction
- Follow-up long enough to assess outcome – Somewhat (12m)
- Excluded populations – Did not meet NHLBI PPCM criteria, echo unavailable, duplicates, missing race, outside UAB
- Bias reported – Transfers; confounding; limited follow-up; small sample; Black/White only; high smoking rate

Tahir

- Comparability / adjusts for – Age, initial EF, BMI, diabetes, hypertension
- End outcome reported – Recovery of EF, myocardial recovery
- Variation in follow-up period – Mean 9.1 ± 10.4 months
- Adequacy of follow-up – Not reported (27 patients at start)
- Follow-up long enough to assess outcome – Somewhat (mean ~9m)
- Excluded populations – Age <18 or >45, ischemic CM, prior heart disease, no follow-up echo
- Bias reported – Small sample; retrospective; single-center; urban population; variable follow-up

Case/Control Gentry

- End outcome: Assess if AA at increased risk of developing PPCM
- Variation in follow-up: 0
- Bias: May not have detected the occurrence of peripartum cardiomyopathy in women who were mildly affected and did not require hospital admission. Possible bias. Hospital based design. Small sample size. Single center, Missing data.

**Supplementary Appendix C: Newcastle-Ottawa Rating Tool**

**NEWCASTLE - OTTAWA QUALITY ASSESSMENT SCALE CASE CONTROL STUDIES**

Note: A study can be awarded a maximum of one star for each numbered item within the Selection and Exposure categories. A maximum of two stars can be given for Comparability.

**Selection**

1) Is the case definition adequate?

a) yes, with independent validation **🟑**

b) yes, eg record linkage or based on self reports

c) no description

2) Representativeness of the cases

a) consecutive or obviously representative series of cases **🟑**

b) potential for selection biases or not stated

3) Selection of Controls

a) community controls **🟑**

b) hospital controls

c) no description

4) Definition of Controls

a) no history of disease (endpoint) **🟑**

b) no description of source

**Comparability**

1) Comparability of cases and controls on the basis of the design or analysis

a) study controls for _______________ (Select the most important factor.) **🟑**

b) study controls for any additional factor **🟑** (This criteria could be modified to indicate specific control for a second important factor.)

**Exposure**

1) Ascertainment of exposure

a) secure record (eg surgical records) **🟑**

b) structured interview where blind to case/control status **🟑**

c) interview not blinded to case/control status

d) written self report or medical record only

e) no description

2) Same method of ascertainment for cases and controls

a) yes **🟑**

b) no

3) Non-Response rate

a) same rate for both groups **🟑**

b) non respondents described

c) rate different and no designation

**NEWCASTLE - OTTAWA QUALITY ASSESSMENT SCALE**

**COHORT STUDIES**

Note: A study can be awarded a maximum of one star for each numbered item within the Selection and Outcome categories. A maximum of two stars can be given for Comparability

**Selection**

1) Representativeness of the exposed cohort

a) truly representative of the average _______________ (describe) in the community **🟑**

b) somewhat representative of the average ______________ in the community **🟑**

c) selected group of users eg nurses, volunteers

d) no description of the derivation of the cohort

2) Selection of the non exposed cohort

a) drawn from the same community as the exposed cohort **🟑**

b) drawn from a different source

c) no description of the derivation of the non exposed cohort

3) Ascertainment of exposure

a) secure record (eg surgical records) **🟑**

b) structured interview **🟑**

c) written self report

d) no description

4) Demonstration that outcome of interest was not present at start of study

a) yes **🟑**

b) no

**Comparability**

1) Comparability of cohorts on the basis of the design or analysis

a) study controls for _____________ (select the most important factor) **🟑**

b) study controls for any additional factor **🟑** (This criteria could be modified to indicate specific control for a second important factor.)

**Outcome**

1) Assessment of outcome

a) independent blind assessment **🟑**

b) record linkage **🟑**

c) self report

d) no description

2) Was follow-up long enough for outcomes to occur

a) yes (select an adequate follow up period for outcome of interest) **🟑**

b) no

3) Adequacy of follow up of cohorts

a) complete follow up - all subjects accounted for **🟑**

b) subjects lost to follow up unlikely to introduce bias - small number lost - > ____ % (select an adequate %) follow up, or description provided of those lost) **🟑**

c) follow up rate < ____% (select an adequate %) and no description of those lost

d) no statement

**Supplementary Appendix D: Descriptive Tables of Included Studies**

## Table 1a. Race Stratified Cohorts – No Follow-up Data, Study Descriptions

| Author | Publication date | Data Years | Study Type | Data source | PPCM definition | Cases | Cohort Type | Mean/Median follow-up |
| --- | --- | --- | --- | --- | --- | --- | --- | --- |
| Afana | 2016 | 2004–2011 | Retrospective cohort | Nationwide Inpatient Sample database | ICD-9-CM 674.50–674.54 during delivery hospitalization | 297 African-American / 506 White | Race Strat | 0 |
| Gambahaya | 2022 | 2016–2018 | Retrospective cohort | National Inpatient Sample database | ICD-10-CM during delivery hospitalization | 3180 Black / 3555 White | Race Strat | 0 |
| Kao | 2013 | 2003–2007 | Retrospective cohort | Hospital records CA, NH, NJ, NY, VM, WV | ICD-9-CM 674.5x during delivery hospitalization | 104 Black / 173 White | Race Strat | 0 |
| Krishnamoorthy | 2016 | 2009–2010 | Retrospective cohort | National Inpatient Sample database | ICD-9-CM 674.50–674.54 not limited to delivery | 1721 Black / 1601 White | Race Strat | 0 |

## Table 1b. Race Stratified Cohorts – No Follow-up Data, Data included

| **Author** | **Data included in analysis** | | | | | | | | | | **MACE definition** |
| --- | --- | --- | --- | --- | --- | --- | --- | --- | --- | --- | --- |
|  | # RF | Control Data | Px Postpartum | EF | Recovery | MACE | Combo MACE/Mort | Mortality | In Hospital Mortality | F/U Mortality |  |
| Afana | 6 | X |  |  |  |  |  | X | X |  |  |
| Gambahaya | 7 | X |  |  |  |  |  |  |  |  |  |
| Kao | 10 | X |  |  |  |  | X |  |  |  | Death, cardiac arrest, heart transplantation, and/or mechanical circulatory support. |
| Krishnamoorthy | 10 |  |  |  |  |  |  | X | X |  |  |

## Table 1c. Race Stratified Cohorts – Follow-up Data, Study descriptions

| Author | Publication date | Data Years | Study Type | Data source | PPCM definition | Cases | Cohort Type | Mean/Median follow-up |
| --- | --- | --- | --- | --- | --- | --- | --- | --- |
| Amos | 2006 | 1990–2003 | Retrospective cohort | Duke University Medical Center | NHLBI/ORD Workshop definition | 28 Black / 21 White | Race Strat | 43 months |
| Cooper | 2012 | 2002–2008 | Prospective cohort | 16 academic centers US, IMAC2 | NHLBI/ORD Workshop definition | 14 Black / 25 White | Race Strat | 26 ± 17 months |
| Goland | 2013 | 1993–2007 | Retrospective cohort | USC, LSU | NHLBI/ORD Workshop definition | 52 Black / 104 White | Race Strat | 19 ± 14 months |
| Harper | 2012 | 2002–2003 | Retrospective cohort | NC Hospital discharge, live birth, mortality db | ICD-9-CM | 50 Black / 33 White | Race Strat | 84 months |
| Irizarry | 2017 | 1986–2016 | Retrospective cohort | University of Pennsylvania Health System | ICD-9-CM 674.5 or echo within 6m | 121 African-American / 99 Non-African American | Race Strat | Within 6m |
| Lewey | 2020 | 1986–2016 | Retrospective cohort | University of Pennsylvania Health System | ICD-9-CM 674.5 or echo within 6m | 104 Black / 89 Nonblack | Race Strat | 42.6 months |
| Mahowald | 2019 | 2000–2011 | Retrospective cohort | University of Michigan | ICD-9-CM | 17 Black / 34 White / 8 Other | Race Strat | 76 ± 7.8 months |
| Mcnamara | 2015 | 2009–2012 | Prospective cohort | 30 US academic centers, IPAC | NHLBI/ORD Workshop definition | 30 Black / 70 White | Race Strat | 12 months |
| Pillarsetti | 2014 | 1999–2012 | Retrospective cohort | Univ. Kansas, Detroit Medical Center | ICD-9-CM 674.50–674.54 | 55 Black / 45 Nonblack (39 Caucasian + 6 Hispanic) | Race Strat | 35 ± 21 months |
| Sinkey | 2020 | 2000–2017 | Retrospective cohort | Univ. Alabama at Birmingham | NHLBI/ORD Workshop definition | 46 Black / 49 White | Race Strat | 12 months |

## Table 1d. Race Stratified Cohorts – Follow-up Data, Data included

| **Author** | **Data included in analysis** | | | | | | | | | | **LVEF Recovery Definition** | **MACE definition** |
| --- | --- | --- | --- | --- | --- | --- | --- | --- | --- | --- | --- | --- |
|  | # RF | Control Data | Px Postpartum | EF | Recovery | MACE | Combo MACE/Mort | Mortality | In Hospital Mortality | F/U Mortality |  |  |
| Amos |  |  |  |  | X | X |  | X |  | X | ≥50% |  |
| Cooper |  |  |  | X | X | X |  |  |  |  | >50% |  |
| Goland | 4 |  | X | X | X |  |  | X |  | X | ≥50% | Cardiac arrest, heart transplantation, mechanical circulatory support, thromboembolic event, serious arrhythmias requiring pacemaker/ AICD insertion |
| Harper |  |  |  |  |  |  |  | X |  | X |  |  |
| Irizarry | 5 |  | X | X | X | X |  | X |  | X | >50% | Heart transplantation, arrhythmia |
| Lewey | 4 |  |  |  |  |  |  |  |  |  |  |  |
| Mahowald |  |  |  |  | X |  | X |  |  |  | >55% | Death, heart transplantation, LVAD, chronic inotrope-dependence |
| Mcnamara | 2 |  |  | X | X |  | X |  |  |  | > 50% | Death, heart transplantation, LVAD |
| Pillarsetti | 8 |  | X | X | X | X |  | X |  | X | >50% | Death, ICD |
| Sinkey | 3 |  | X | X | X | X |  | X |  | X | ≥55% | Heart transplantation, LVAD, ICD, Ventilator, Hemodialysis |

## Table 1e. Predominantly Black Cohorts, Study descriptions

| Author | Publication date | Data Years | Study Type | Data source | PPCM definition | Cases | Cohort Type | Mean/Median follow-up |
| --- | --- | --- | --- | --- | --- | --- | --- | --- |
| Briasoulis | 2016 | 2009–2014 | Retrospective cohort | Detroit Medical Center | NHLBI/ORD Workshop definition | 45 African American / 2 Caucasian | Predom Black | 12.5 months (1–60) |
| Chapa | 2005 | 1988–2001 | Retrospective cohort | University of Chicago | ICD-9-CM, FS <30%, LVEDD ≥4.8 cm | 25 African American / 7 White | Predom Black | 46 months |
| Gentry | 2010 | 2003–2008 | Retrospective case-control | Medical College of Georgia | NHLBI/ORD Workshop definition | 26 African American / 2 White | Predom Black | 0 months |
| Lindley | 2017 | 2004–2014 | Retrospective cohort | Barnes-Jewish Hospital | NHLBI/ORD Workshop definition | 30 Black / 9 White | Predom Black | 12 months |
| Modi | 2009 | 1992–2003 | Retrospective cohort | LSU Health Science Center, Shreveport | NHLBI/ORD Workshop definition | 39 African American / 5 White | Predom Black | 24 months (0.1–264) |
| Peters | 2018 | 1992–2016 | Retrospective cohort | Temple Univ. Hospital (Philadelphia, PA) | ICD-9-CM | 40 Black / 6 White / 4 Hispanic / 3 Unknown | Predom Black | 43 months (17–88) |
| Tahir | 2015 | 1998–2012 | Retrospective cohort | Boston Medical Center Ambulatory Clinic | NHLBI/ORD Workshop definition | 20 Black / 7 White | Predom Black | 9.1 ± 10.4 months |

## Table 1f. Predominantly Black Cohorts, Data Included

| **Author** | **Data included in analysis** | | | | | | | | | | **LVEF Recovery Definition** |  |
| --- | --- | --- | --- | --- | --- | --- | --- | --- | --- | --- | --- | --- |
|  | # RF | Control Data | Px Postpartum | EF | Recovery | MACE | Combo MACE/Mort | Mortality | In Hospital Mortality | F/U Mortality |  |  |
| Briasoulis | 5 |  |  | X | X | X |  | X |  | X | >50% |  |
| Chapa | 3 |  | X |  | X |  |  | X |  | X | NA, FS ≥30%, LVEDD <4.8cm | Death, heart transplantation |
| Gentry | 6 | X |  | X |  |  |  |  |  |  | NA |  |
| Lindley | 5 |  | X |  | X |  |  | X |  | X | ≥50% with an absolute improvement of ≥10% |  |
| Modi | 4 |  | X | X | X |  |  | X |  | X | ≥50% |  |
| Peters | 3 |  |  | X | X | X |  | X |  | X | ≥50% | Death, heart transplantation, LVAD |
| Tahir | 6 |  |  | X | X |  |  |  |  |  | ≥50% |  |
